# Supplementary material for: The Significance of Phenotyping and Quantification of Plasma Extracellular Vesicles Levels Using High-Sensitivity Flow Cytometry during COVID-19 Treatment
Source: Viruses. 2021 Apr 27;13(5):767. doi: 10.3390/v13050767 (PMC8146052; doi:10.3390/v13050767)
Supplement: Supplementary file 1 [file viruses-13-00767-s001.zip › viruses-1167777-supplementary.pdf]

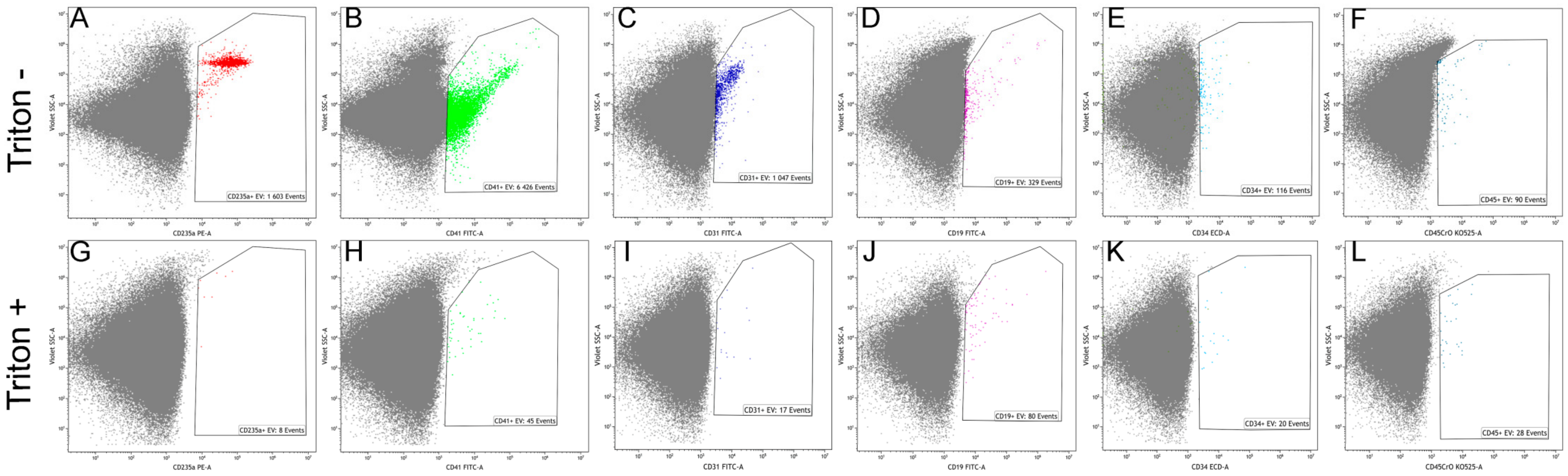

Supplementary Figure S1. Detergent sensitivity of the immunostained events performed using high-sensitivity flow cytometer Cytoflex S. Upper row represents results before and bottom row – after Triton X100 treatment. To make the figures more representative, all coupled pictures (before and after Triton X100) were normalized to experiment acquisition time. A, G. CD235a+ EV. B, H. CD41+ EV. C, I. CD31+ EV. D, J. CD19+ EV. E, K. CD34+ EV. F, L. CD45+ EV.
